# Generating Ensembles of Gene Regulatory Networks to Assess Robustness of Disease Modules

**DOI:** 10.3389/fgene.2020.603264

**Published:** 2021-01-14

**Authors:** James T. Lim, Chen Chen, Adam D. Grant, Megha Padi

**Affiliations:** ^1^Department of Molecular and Cellular Biology, The University of Arizona, Tucson, AZ, United States; ^2^Department of Epidemiology and Biostatistics, Mel and Enid Zuckerman College of Public Health, The University of Arizona, Tucson, AZ, United States; ^3^University of Arizona Cancer Center, The University of Arizona, Tucson, AZ, United States

**Keywords:** network, community significance, community robustness, network community, community detection, regulatory network, community structure, cancer

## Abstract

The use of biological networks such as protein–protein interaction and transcriptional regulatory networks is becoming an integral part of genomics research. However, these networks are not static, and during phenotypic transitions like disease onset, they can acquire new “communities” (or highly interacting groups) of genes that carry out cellular processes. Disease communities can be detected by maximizing a modularity-based score, but since biological systems and network inference algorithms are inherently noisy, it remains a challenge to determine whether these changes represent real cellular responses or whether they appeared by random chance. Here, we introduce Constrained Random Alteration of Network Edges (CRANE), a method for randomizing networks with fixed node strengths. CRANE can be used to generate a null distribution of gene regulatory networks that can in turn be used to rank the most significant changes in candidate disease communities. Compared to other approaches, such as consensus clustering or commonly used generative models, CRANE emulates biologically realistic networks and recovers simulated disease modules with higher accuracy. When applied to breast and ovarian cancer networks, CRANE improves the identification of cancer-relevant GO terms while reducing the signal from non-specific housekeeping processes.

## Introduction

Finding the underlying molecular mechanisms that drive complex disease remains a difficult problem. Complex diseases appear to be caused by many perturbations scattered around the gene regulatory network, which creates a considerable amount of variability in disease susceptibility (Schadt et al., 2009; Califano et al., 2012; Pickrell, 2014). Network analysis has therefore become a popular approach to model molecular interactions in the cell and prioritize candidate disease genes (Greene et al., 2015; Marbach et al., 2016; Santolini and Barabasi, 2018). Many of these methods capitalize on the idea that biological networks are composed of “communities,” or modules, of genes that work in concert to carry out cellular functions and cause a disease (Hartwell et al., 1999; Menche et al., 2015; Platig et al., 2016). A module in a biological network typically refers to a set of genes that is densely interconnected in the network, function together, or are co-regulated (Girvan and Newman, 2002; Segal et al., 2003; Ghiassian et al., 2015). Identifying the changes in network structure associated with disease onset can reveal more mechanistic insights than standard approaches like differential expression analysis; this approach is often called “differential network biology” (Ideker and Krogan, 2012). A wide variety of tools have been developed to identify the changes in network edges and network structure that accompany disease (Gill et al., 2010; Tesson et al., 2010; Gambardella et al., 2013; Van Landeghem et al., 2016).

However, evaluating the robustness and significance of changes in network structure remains a challenge. Gene regulatory networks are often inferred from transcriptomic data using imperfect inference tools, with no easy way of assessing their underlying variance (Lancichinetti et al., 2011; Menche et al., 2015; Choobdar et al., 2019; Palowitch, 2019). Moreover, community detection algorithms can lead to multiple solutions corresponding to local optima of the fitness function (Newman, 2006; Blondel et al., 2008; Campigotto et al., 2014). Two types of approaches are often used to judge the quality of network communities: consensus clustering and statistical significance (Lancichinetti et al., 2011; Lancichinetti and Fortunato, 2012; Menche et al., 2015; Zitnik and Leskovec, 2018; Palowitch, 2019). The consensus approach combines multiple solutions from the optimization algorithm to find the most likely assignment of genes to communities (Lancichinetti and Fortunato, 2012; Choobdar et al., 2019). Alternatively, the statistical significance of individual communities can be estimated by comparing them with a null distribution derived from randomized networks with the same degree characteristics as the original network (Ideker et al., 2002; Emmert-Streib, 2007; Lancichinetti et al., 2011; Mall et al., 2017; Kojaku and Masuda, 2018; Newman, 2018). Network randomization is typically carried out using generative models.

In the present study, we set out to rank the most robust disease-driven changes in the community structure of gene regulatory networks. We first inferred weighted bipartite networks by integrating transcription factor (TF) binding motifs and gene expression data, and then optimized a modularity-based score to identify candidate modules more active in disease conditions than in matched controls (Padi and Quackenbush, 2018). Other approaches for differential network analysis could be used, including DiffCoEx, DINA, DNA, and Diffany (Gill et al., 2010; Tesson et al., 2010; Gambardella et al., 2013; Van Landeghem et al., 2016), but these methods are limited to either identifying individual correlation-based edges or examining pre-defined gene sets and network features, making them less generalizable to multiple types of questions and networks. Modularity optimization methods can help reveal new biological insights across multiple contexts, but they typically result in multiple solutions and cannot provide information about which disease modules are the most robust or significant.

We tried applying existing methods to rank the most significant genes within our candidate disease modules. Consistent with previous observations, consensus clustering led to a loss of resolution and an inability to detect smaller gene sets annotated to more informative biological pathways (Lancichinetti and Fortunato, 2012; Jeub et al., 2018). Next, we estimated the significance of the disease modules relative to a null distribution for the control network created using two popular generative models – the configuration model (Gabrielli et al., 2019) and the stochastic block model (SBM) (Aicher et al., 2015). However, these models could not realistically simulate the characteristics of a gene regulatory network. Transcriptional regulation is strongly constrained by the fact that any given TF regulates a limited number of genes, depending on TF binding sites, activators/repressors, and epigenetic state (Roeder, 1996; Ptashne and Gann, 1997; Lee and Young, 2000; Teif and Rippe, 2009; Gerstein et al., 2012). Both the configuration model and SBM ignore this restriction and assume that each TF node can influence all genes in the network (configuration) or all genes in a community (SBM), which leads to improper sampling of edge weight variance.

Therefore, we identified a need for a new, computationally efficient generative model that accounts for the known constraints of gene regulation (Proulx et al., 2005; Bansal et al., 2009; Sah et al., 2014; Fosdick et al., 2018). It is challenging to randomize weighted networks while imposing multiple constraints, because each modification propagates to the rest of the network, leading to extreme edge weights if they are not properly controlled. There is no accepted method for generating ensembles of weighted bipartite networks with fixed node strengths (the total weight of edges adjoining each node) (Fosdick et al., 2018). Here we present a new algorithm for network randomization called Constrained Random Alteration of Network Edges (CRANE). CRANE can produce ensembles of unipartite or bipartite weighted networks with fixed node strengths that resemble gene regulatory networks. These ensembles can be used as null distributions to evaluate the importance of genes and regulators in candidate disease modules. To demonstrate the utility of CRANE, we apply it to simulated disease modules, as well as transcriptional networks derived from angiogenic ovarian tumors and hormone receptor-positive breast cancers. In simulations, CRANE performs better than all comparable approaches in finding the “true” disease module. When applied to breast and ovarian cancer networks, several methods are able to improve identification of cancer-related processes in specific cases, but CRANE is the only one that consistently reveals biological insights across multiple networks and conditions while also reducing background noise from non-specific housekeeping processes. Our study demonstrates that CRANE can evaluate candidate disease modules to identify a subset of genes that is robustly associated to the disease.

## Materials and Methods

### General Workflow

**FIGURE 1:**
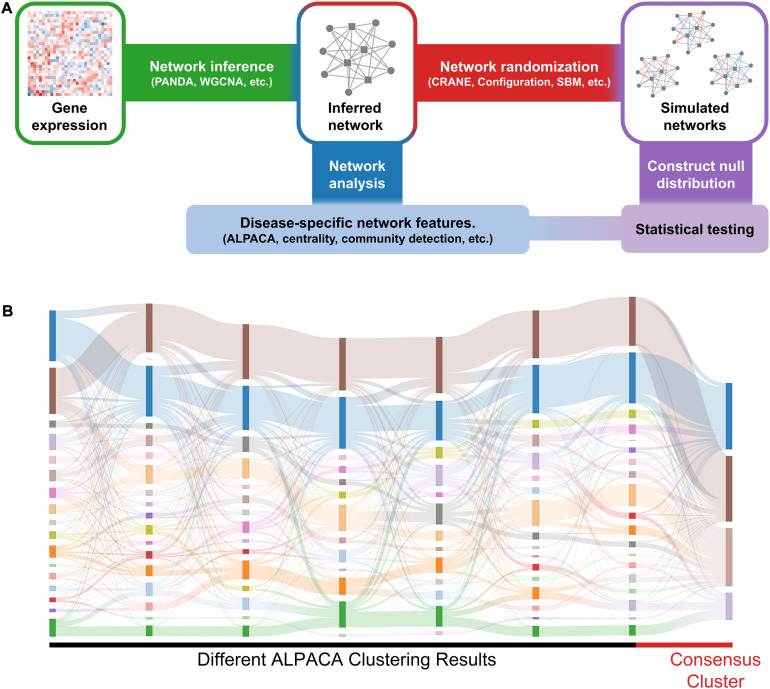
General workflow and results of consensus clustering. **(A)** We identify significant changes in network structure by comparing disease-specific network features against a null distribution. We first construct disease-specific and matched control networks from gene expression data (e.g., RNA-seq) using a network inference algorithm. Next, we compare the networks to extract disease-specific network features. Independently, we apply network randomization to the control network to create a null distribution. The disease-specific network features are then compared against the null distribution to evaluate statistical significance. **(B)** The Sankey plot shows community assignments from seven separate runs of ALPACA on angiogenic vs. non-angiogenic ovarian cancer networks. Each column represents an ALPACA solution with the far-right column showing the result from consensus clustering of 1,000 different ALPACA solutions. The height of each box and ribbons indicates the size of each module and the number of shared nodes between corresponding modules, respectively.

To rank significant nodes in disease modules, we use the following general procedure (Figure 1A): we first construct disease and matched control networks from gene expression data (e.g., RNA-seq) using a *network inference algorithm*. Next, we identify disease-specific network features (e.g., disease modules) using *network analysis methods*. Our main goal is to evaluate the significance of these disease-specific features. To do this, we compare their associated scores in the disease network to a null distribution created from the control network using a *network randomization algorithm*. For large data sets (*n* > 300), a “true” null distribution can be generated by subsetting the expression profiles for the matched controls (*n* = 50 for each subsample) and constructing independent “replicate” control networks.

### Using CRANE or Other Methods to Evaluate Disease Modules: Basic Procedure

Here, we describe the basic procedure for ranking significant genes in disease modules using network randomization algorithms. We provide callouts to other subsections of Section “Materials and Methods” where more details can be found.

We first create disease and matched control networks, either from gene expression data using a *network inference algorithm* (see “Data preprocessing and network inference”), or by simulating networks with artificial disease modules (see “Simulated networks”). Second, we compare the two networks using ALPACA with default parameters, as implemented in the R package (freely available^1^), to identify candidate disease modules (Padi and Quackenbush, 2018). We choose to use ALPACA, but one can use any network analysis tool that groups nodes together, including standard community detection techniques like modularity maximization and differential analysis tools like DiffCoEx, DINA, DNA, and Diffany (Gill et al., 2010; Tesson et al., 2010; Gambardella et al., 2013; Van Landeghem et al., 2016). ALPACA outputs a vector consisting of a module assignment $K_{\text{node}}^{0}$ and differential modularity score $S_{\text{node}}^{0}$ for each node. $S_{\text{node}}^{0}$ quantifies how much the node contributes to the global change in modularity between the disease and control networks. Next, we use a *network randomization algorithm* – either CRANE with α in the range of 0.1 to 0.4 (see section “CRANE Algorithm”), SBM (see section “Stochastic Block Model”), configuration model (see section “Configuration Model”), random edge weight permutation (see section “Random Edge Weight Permutation”) or data subsampling – on the control network (*N*^0^)to obtain a null distribution for $S_{\text{node}}^{0}$. To do this, we perform the following steps:

(i) Use *network randomization algorithm* to generate a perturbed network (*N*^P^) starting from the control network (*N*^0^).
(ii) Compute a “null” differential modularity matrix $D_{\text{ij}}^{\text{P}}$ for each *N*^P^ by comparing *N*^P^ to the original control network *N*^0^ using an in-built function in the ALPACA R package.
(iii) Score each node according to its contribution to the differential modularity $D_{\text{ij}}^{\text{P}}$ of the module defined by $K_{\text{node}}^{0}$, to get its score $S_{\text{node}}^{\text{P}}$ under the null hypothesis that the observed change in network structure is only due to measurement noise.

We repeat (i)–(iii) for 1,000 perturbed networks and use the resulting vector of $S_{\text{node}}^{\text{P}}\,$ values to fit a null distribution *T*_*node*_ for each node.Finally, we compute the *p*-value for each node (i.e., its significance as a member of a true disease module) by assuming *T*_*node*_ follows a normal distribution:

$$P-v\,a\,l\,u\,e=1-\text{Φ}\,\left(\frac{S_{\text{node}}^{0}-m\,e\,a\,n\,\left(T_{\text{node}}\right)}{s\,d\,\left(T_{\text{node}}\right)}\right)$$

where Φ is the normal cumulative distribution function, and *mean* and *sd* represent the mean and standard deviation of the node score distribution. To evaluate the results of each network randomization algorithm, we ranked all the genes in the network by their *p*-value and either statistically compared this ranking against the true disease genes (in the case of simulated networks; see section “Simulated Networks”), or evaluated the top-ranked genes within each module for functional enrichment (in the case of real cancer data). In the latter case, in order to make our conclusions threshold-independent, we evaluated the top 25, 50, 75, etc., up to 500 core genes (for example, in one typical module, this would correspond to genes with adjusted *p*-values less than 10^–23^, 10^–21^, 10^–19^, etc. up to 0.01) from each module to identify enriched GO terms at different cutoffs. All significant GO terms (*P*_*adj*_ < 0.05) across all cutoffs were included in the final result for each module (see “Module-Specific Functional Enrichment Analysis” section below for more details). GO term enrichment was calculated using the R package GOstats (v2.54.0), with the following parameters: the gene universe is the set of all possible target genes in the initial networks and the *p*-value calculation is conditioned on the GO hierarchy structure. In each module, the GOstats *p*-values were adjusted for multiple testing using the Benjamini–Hochberg method. We note that all genes can be ranked together by their *p*-values (with adjusted *p*-value < 0.05 as significant) to combine signals from all modules across the whole network, or top-ranked genes from each module can be kept separate and interpreted as smaller sets of tightly interacting genes.

In addition to this basic procedure, we motivated the development of CRANE by performing consensus clustering on multiple stochastic runs of ALPACA, which uses the Louvain method for community detection (see section “Consensus Clustering” for more details). We also quantified the similarity between networks created by CRANE by computing the normalized mutual information (see section “Computing NMI” for more details).

### Module-Specific Functional Enrichment Analysis

The following steps were taken to evaluate and compare the performance of each network randomization method at uncovering module-specific disease-relevant GO terms in cancer networks:

(i) Take all genes assigned to one module and rank them by their network randomization score (e.g., CRANE *p*-value).
(ii-a) Extract the top 25 genes (e.g., genes with CRANE-derived adjusted *p*-value < 10^–23^) and compute the adjusted *p*-value for overlap with a disease relevant GO term (e.g., “blood vessel development” for angiogenic ovarian cancer) using a hypergeometric test.
(ii-b) Repeat (ii-a) with top 50 genes (e.g., genes with CRANE-derived adjusted *p*-value < 10^–21^).
(iii) Repeat (ii) iteratively until top 500 genes (e.g., genes with CRANE-derived adjusted *p*-value < 0.01).
(iv) All GO term *p*-values across all thresholds (20 *p*-values per GO term) are collected and the average of the corresponding −1*log_10_ *p*-value is reported.

### Data Preprocessing and Network Inference

Batch-corrected and normalized ovarian PanCancer TCGA RNA-seq values were downloaded from cBioPortal (Cancer Genome Atlas Research Network, 2011; Cerami et al., 2012; Gao et al., 2013). Low-expressing genes were removed by keeping only genes with at least 1 count per million in at least half of the total samples using the R package edgeR (v3.26.5) and processed with the voom function within the R package limma (v3.38.3) using TMM normalization. Angiogenic (*n* = 124) and non-angiogenic (*n* = 166) tumors were grouped as described in Glass et al. (2015). Preprocessed METABRIC breast cancer expression data was downloaded from cBioPortal (Cerami et al., 2012; Curtis et al., 2012; Gao et al., 2013; Pereira et al., 2016), along with estrogen receptor negative (ER−; *n* = 445) and estrogen receptor positive (ER+; *n* = 1449) status as measured by immunohistochemistry.

Many methods are available to infer gene regulatory networks from transcriptomic data, including ARACNE, CLR, MERLIN, PANDA, and WGCNA, but there is no clear winner across all contexts (Zhang and Horvath, 2005; Margolin et al., 2006; Marbach et al., 2012; Glass et al., 2013; Zhang et al., 2016; Siahpirani and Roy, 2017). For our analyses we chose to use PANDA (Passing Attributes between Networks for Data Assimilation) and WGCNA.

#### PANDA

We chose to use PANDA to construct our gene regulatory network because it can integrate known transcription factor (TF) binding sites, and because it does not use TF mRNA level as a proxy for TF activity, instead inferring this latent variable from target gene co-expression, making it particularly appropriate for mammalian contexts where TFs are often regulated by post-translational modification, competitive binding, or localization. Expression data from each subtype was integrated with transcription factor binding sites using the network inference algorithm PANDA with default parameters to create subtype-specific regulatory networks (Glass et al., 2013). Subsampled networks were inferred by selecting random subsets of 50 subjects without replacement from the gene expression of each respective subtype. A prior network of binding sites for 730 TFs was defined as the occurrence of the corresponding motif in a [−750,+250] bp window around the transcription start site (Sonawane et al., 2017). The following formula was applied for analyses requiring exponentially transformed PANDA edge weights (Sonawane et al., 2017), where *w*_*ij*_ are the initial *z*-score edge weights output by PANDA, and *W*_*ij*_ are the final transformed edge weights:

$$W_{\text{ij}}=\ln\left(e^{{\text{w}}_{\text{ij}}}+1\right)$$

#### WGCNA

We constructed signed weighted gene co-expression networks using the R package WGCNA (v1.69) (Zhang and Horvath, 2005). Input for the co-expression network consisted of normalized expression values from 1,000 randomly selected genes and random subsets of 50 subjects chosen without replacement from the ER+ METABRIC breast cancer expression data. For all subsampled WGCNA networks, a soft thresholding power of eight was used.

### CRANE Algorithm

CRANE takes a weighted network as input and provides a perturbed version of that network as output. In the following, we will describe the procedure for bipartite networks. We first compute the strength of node *i* as the sum of the edge weights adjoining that node, or $S_{\text{i}}=\sum_{j}w_{\text{ij}}$ (As an optional step to increase network variance further, noise can be added to the original sequence of node strengths by adding normally distributed random numbers with mean 0 and standard deviation estimated from subsampled networks). Given *m* is the total number of TFs and *n* is the total number of genes, *A*_*ij*_ is the *m* × *n* adjacency matrix of the input network where rows (TFs) and columns (genes) are ordered randomly. We create an empty *m* × *n* adjacency matrix *B*_*ij*_ that will become the perturbed network. The first row (first TF) of *B*_*ij*_ is initialized with edge weights from the first row of *A*_*ij*_. Then for each *TF*_*l*_, where *l* = [1,…,*m*−1], we apply the following steps: we perturb the current (*l*th) row *B*_*lj*_ by adding normally distributed random numbers with mean 0 and standard deviation computed from the original edge weights for *TF*_*l*_, i.e., *sd*(*A*_*l*j_). This perturbation is multiplied by a parameter α, giving the user the ability to adjust the magnitude of the perturbation. The *B*_*lj*_ edge weights are multiplied by a factor of $\,\sum_{j=1}^{n}A_{\text{lj}}/\sum_{j=1}^{n}B_{\text{lj}}$ to ensure the TF strength in *B*_*lj*_ is equal to *A*_*lj*_. We compute initial values for *B*_*l+1,j*_ (edge weights for the next TF) by computing $\sum_{i=1}^{l+1}A_{\text{ij}}-\sum_{i=1}^{l}B_{\text{ij}}$, thus keeping the node strengths in *B*_*ij*_ equal to the node strengths in *A*_*ij*_. After the initial *B*_*l+1,j*_ have been determined, we check if any edge weights within *B*_*l+1,j*_ fall outside of the global maximum or minimum of the original edge weights in *A*_*ij*_. For any values in *B*_*l+1,j*_ greater than *max*(*A*_ij_) edge weight, we add the difference in value between *B*_*l+1,j*_ and *max*(*A*_ij_) to the corresponding *B*_*lj*_. For any values *B*_*l+1,j*_ less than *min*(*A*_ij_) we subtract the difference in value between *B*_*l+1,j*_ and *min*(*A*_ij_) to the corresponding *B*_*lj*_. Then the modified edge weights in *B*_*lj*_ are normalized to maintain the correct TF strength and a new set of *B*_*l+1,j*_ are computed. We repeat the correction process until all values are within the range of *A*_*ij*_.

Note that α is the only user-adjustable parameter in CRANE; in the Results section, we provide a robustness analysis and guidance for choosing an appropriate value of α. A more detailed description of CRANE, including pseudocode, can be found in the Supplementary Methods. A unipartite version of CRANE is also available for use. CRANE is freely available as an R package at https://github.com/PadiLab/CRANE.

### Configuration Model

The configuration model is a method for generating random networks from a given node degree or strength sequence (Garlaschelli, 2009; Mastrandrea et al., 2014; Gabrielli et al., 2019). For weighted networks, the configuration model is typically constructed as an exponential random graph. To fit the configuration model to the PANDA network, we transformed z-score edge weights to positive weights using the formula given in the “Data Preprocessing and Network Inference” section. Based on the fact that PANDA is a fully connected graph, the configuration model can be written as described in Gabrielli et al. (2019), i.e.,

$$P\,\left(A\right)=\prod_{\text{ij}}e^{-\left(\theta_{\text{i}}+\theta_{\text{j}}\right)\,a_{\text{ij}}}\,\left(\theta_{\text{i}}+\theta_{\text{j}}\right)$$

where *P* is the probability of a network with adjacency matrix *A* and edge weights given by its entries *a*_*ij*_, and the θ parameters are Lagrange multipliers that need to be estimated. The Maximal Likelihood (ML) function then constrains the θ parameters by the given node strength sequence:

$$\sum_{\text{j}}{\left(\theta_{\text{i}}+\theta_{\text{j}}\right)}^{-1}=\sum_{\text{j}}a_{\text{ij}}=S_{\text{i}}$$

where *S*_*i*_ represents the strength of node *i*, which is defined as the summation of edge weights adjoining node *i*. We used Barzilai–Borwein spectral methods for directly solving this ML system of equations using the R package *BB* (v 2019.10.1) (Varadhan and Gilbert, 2009).

### Stochastic Block Model

The stochastic block model (SBM) is a random graph generative model. The SBM defines a probability distribution over networks by assuming pre-existing communities or “blocks” where the intracommunity edges are stronger (larger edge weights) than intercommunity edges. This probability distribution can be used to produce random graphs with pre-defined inter- and intra-community edge densities. Fitting a stochastic block model (SBM) to the PANDA network is very time consuming (Aicher et al., 2015). To efficiently test the performance of SBM, we introduced a strong assumption of equivalence between modularity optimization and SBM maximum likelihood (Newman, 2016). Thus, the network community structure found by CONDOR (Complex Network Description of Regulators) (Platig et al., 2016) – a modularity maximization method for weighted bipartite networks – was directly used to generate the block structure in the SBM. We assumed a normal distribution for the edge weights as the PANDA network edge weights represent z-scores. The parameters for every block can then be estimated directly using the sample mean and sample variance of the corresponding edge bundles.

### Random Edge Weight Permutation

We wanted to compare CRANE against a naïve method of randomizing a gene regulatory network by permuting its edge weights. Fully permuting the network leads to unrealistic results due to destruction of prior motif information and community structure. To retain as much of the prior biological information as possible, the edges in the network were first divided into motif-positive and motif-negative groups based on whether they were included in the prior network of binding sites for 730 TFs. Next, communities were detected using CONDOR (Platig et al., 2016). Finally, the inter- and intra-community edge weights were grouped together by motif status and randomly shuffled.

### Simulated Networks

To simulate disease modules, we first took a random subset of 50 subjects out of 445 subjects from the estrogen receptor negative (ER-) METABRIC breast cancer expression data and constructed a baseline PANDA network. We then inserted high edge weights (edge weight = 5) between randomly selected TFs and genes to create a simulated disease network. The new module consisted of between 3 and 20 TFs, and five times as many genes as TFs. The simulated disease network was compared to a second “replicate” baseline network inferred from an independent random subset of 50 subjects from the ER- breast tumors. We applied a panel of methods – including ALPACA, consensus clustering, CRANE (α = 0.1–0.4), configuration model, SBM, and random edge weight permutation – and evaluated the results of each method by comparing the ranks of true positives (the known genes in the disease module) against a background consisting of genes not in the disease module. Kolmogorov–Smirnov and Wilcoxon rank-sum tests were used to compute the *p*-value for the difference in the distribution of the ranks. Both tests gave similar results, and so in the figures, we present the Wilcoxon *p*-values. *F*-scores were also computed to evaluate the accuracy of each method using the following formula:

$$F=\frac{T\,r\,u\,e\,P\,o\,s\,i\,t\,i\,v\,e\,s}{T\,r\,u\,e\,P\,o\,s\,i\,t\,i\,v\,e\,s+0.5\,\left(F\,a\,l\,s\,e\,P\,o\,s\,i\,t\,i\,v\,e\,s+F\,a\,l\,s\,e\,N\,e\,g\,a\,t\,i\,v\,e\,s\right)}$$

Positives were defined as the top 1% of ranked nodes.

### Consensus Clustering

To generate consensus clusters, we first repeated ALPACA 1,000 times on the same pair of transcriptional networks, as described in Padi and Quackenbush (2018) but with the *n* nodes ordered randomly in each iteration of the Louvain algorithm. We combined the 1,000 resulting partitions to create an *n* × *n* consensus matrix *C* with each entry*C*_ij_ indicating the number of partitions in which nodes *i* and *j* of the network were assigned to the same cluster, divided by the total number of partitions (1,000). For the final step, we applied the Louvain algorithm (R package igraph v1.2.4.1) on *C* to find the consensus cluster membership for each node (Csardi and Nepusz, 2006; Blondel et al., 2008).

### Computing NMI

The algorithm CONDOR with default parameters was used to detect the community structure of weighted bipartite networks (Greene et al., 2015; Platig et al., 2016). Using CONDOR community assignments as input, the normalized mutual info (NMI) score between two networks was computed using the “compare” function in the R package igraph (v1.2.4.1) (Danon et al., 2005; Csardi and Nepusz, 2006).

### ALPACA and CRANE Pipeline Implementation

To implement the ALPACA and CRANE analysis pipeline presented, first gene regulatory networks for the disease and the control conditions should be inferred from gene expression using PANDA (Glass et al., 2013). The “alpaca.crane” function within the R package CRANE^2^ will automatically run ALPACA (also available separately^3^) to compare the two networks and output the module membership and the significance of the nodes.

## Results

### Existing Methods for Evaluating Significance of Disease Modules

We tried applying the most popular available methods for identifying significant changes in community structure – namely, consensus clustering and comparing against randomized networks – on cancer networks. To apply these methods, we first need to define the networks and candidate disease modules (Figure 1A). PANDA was applied as described in the “Materials and Methods” section (“Data preprocessing and network inference”) to TCGA data from angiogenic and non-angiogenic ovarian tumors (Cancer Genome Atlas Research Network, 2011) and to METABRIC breast cancer data (Curtis et al., 2012; Pereira et al., 2016) to produce weighted bipartite networks.

We next needed to find a set of candidate disease modules, or groups of genes that interact more with each other in one cancer subtype than expected. To do this, we used ALPACA, a method we previously developed that optimizes a differential modularity score (DMS) to identify groups of nodes exhibiting higher inter-node connectivity in a disease (e.g., angiogenic) network than in a matched control (e.g., non-angiogenic) network (Padi and Quackenbush, 2018). Although we chose to use PANDA and ALPACA (other choices are described in section “Materials and Methods”), we note that the following analyses – including consensus clustering, comparison against randomized networks, and CRANE – can be carried out for any networks (inferred using any method) and any subset of nodes (or disease module) that have stronger interactions in the disease network than in the matched control.

To implement consensus clustering, we merged one thousand partitions from individual ALPACA solutions derived by comparing angiogenic vs. non-angiogenic ovarian tumor data to generate a consensus co-membership matrix (see section “Materials and Methods” for details). We then applied the Louvain method to the consensus matrix to determine consensus community assignment (Blondel et al., 2008). Consistent with previous observations in the literature, we found that consensus clustering led to a significant loss of resolution (Figure 1B) (Lancichinetti and Fortunato, 2012; Choobdar et al., 2019) and the inability to detect more specific disease pathways with richer biological interpretations (Jeub et al., 2018).

**FIGURE 2:**
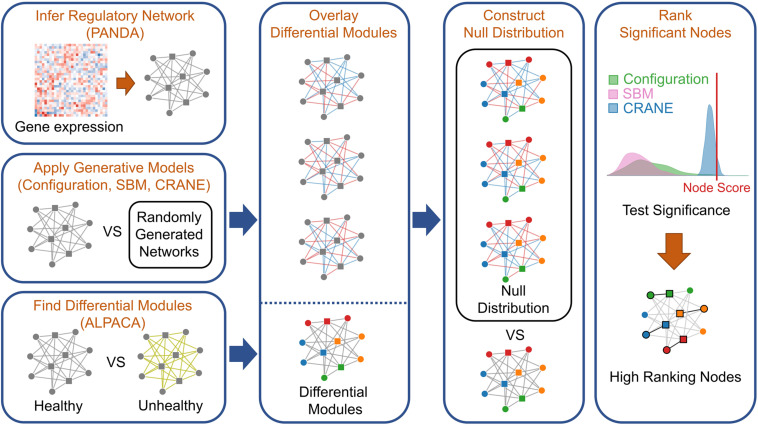
Workflow for applying network generative models to rank genes in disease modules. To integrate ALPACA with generative models (network randomization), we first construct transcriptional networks representing the “control” and “disease” networks by integrating known TF binding sites with gene expression data. We then use ALPACA to identify putative disease modules and compute the differential modularity scores (DMS) for each node. We construct the null distribution of the DMS by comparing the control network to randomized networks generated using the configuration model, SBM, permutation, or CRANE. The *p*-value is calculated by comparing the true node DMS to the null distribution.

Next, we used leading network generative models to create a null distribution by randomizing the control network, against which we can compare the disease module scores and estimate their significance. We chose the configuration model (Gabrielli et al., 2019) and the stochastic block model (SBM) (Aicher et al., 2015) as they both have rigorous mathematical descriptions and are two of the most commonly used generative models (Saul and Filkov, 2007; Sah et al., 2014; Baum et al., 2019). The configuration model constrains the expectation value of the node strengths to match the original network, and assumes an exponential distribution for the edge weights (Garlaschelli, 2009; Mastrandrea et al., 2014; Gabrielli et al., 2019). The stochastic block model defines a probability distribution over networks by matching the pre-existing communities or “blocks” of closely connected nodes found in the original network (Aicher et al., 2015). To evaluate the accuracy of these generative models, we chose to analyze METABRIC breast cancer expression data, one of the few diseases in which there are enough expression profiles to subsample eight independent sets of 50 baseline (ER−) expression profiles and generate “biological replicate” PANDA networks. We applied ALPACA to compare ER+ vs. ER− tumors and identify candidate ER+ modules. We next constructed a “true” null distribution of differential modularity scores for each gene in the candidate modules using the eight “biological replicate” networks. We then compared the characteristics of this true null against ensembles of randomized ER- networks produced by SBM and the configuration model (Figure 2; see section “Materials and Methods” for details).

**FIGURE 3:**
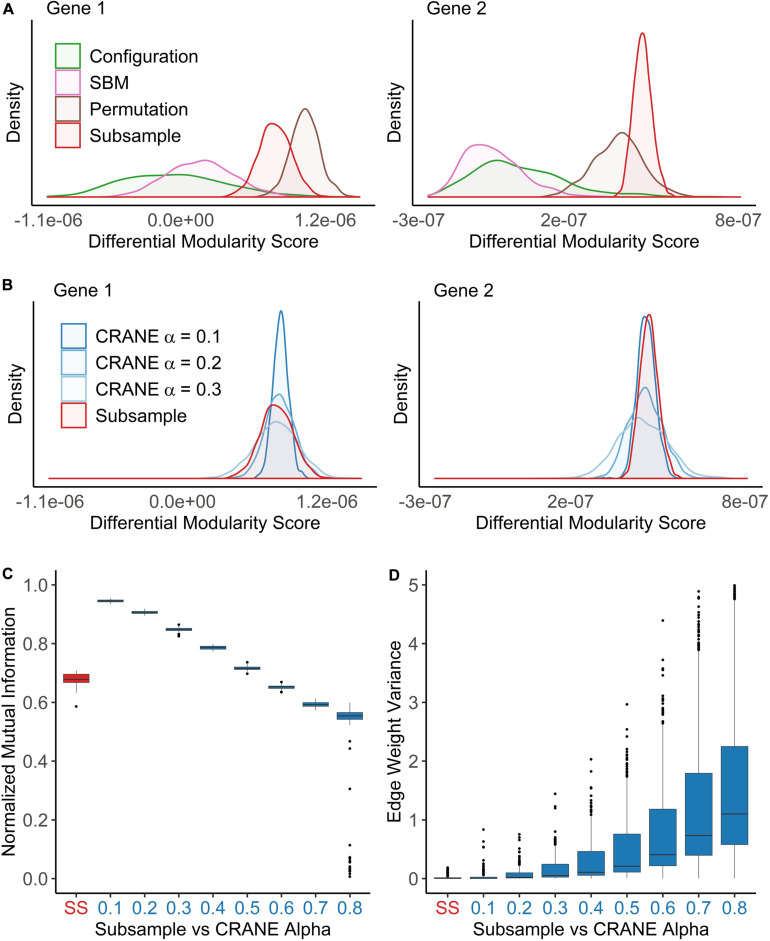
CRANE can generate networks that resemble subsampled data while maintaining control of key network properties. Using the breast cancer transcriptional network as a reference, network ensembles were generated using configuration model, SBM, permutation, and CRANE. As a “true” null distribution, eight PANDA networks were inferred by subsampling (*n* = 50) the gene expression data without replacement from the estrogen receptor negative subtypes. **(A,B)** Density plots showing the null distribution of the differential modularity score (*x*-axis) computed using different methods for two example genes. **(C,D)** The boxplots show the impact of the CRANE alpha parameter on **(C)** community structure and **(D)** edge weight variance, as compared to subsampled (SS) networks. **(C)** Plot showing the normalized mutual information (*y*-axis) between the reference network and CRANE-generated networks for different values of alpha (*x*-axis). **(D)** The edge weight variance (*y*-axis) among subsampled or CRANE-generated networks at different values of alpha (*x*-axis).

We found that both SBM and the configuration model failed to accurately recapitulate the true null distribution computed from the subsampled networks (Figure 3A). Both methods appear to overestimate the edge weight variance, probably because they ignore the physical constraints (e.g., TF binding motifs or chromatin accessibility patterns) by which cells specify patterns of gene regulation (Aicher et al., 2015; Gabrielli et al., 2019). To check whether this observation could hold more generally, we repeated the analysis using a different, commonly used network inference method called WGCNA (weighted gene co-expression network analysis) which generates a matrix of gene co-expression values and applies soft-thresholding to impose a scale-free topology criterion (Zhang and Horvath, 2005). This thresholding procedure converts the co-expression to a new value that can be interpreted as a connection weight. We found that the configuration model also fails to fit subsampled WGCNA breast cancer networks (Supplementary Figure 1), likely because it puts equal emphasis on all the edges and does not properly conserve the highest-confidence regulatory interactions.

### CRANE: New Method for Sampling Weighted Networks

We developed a new algorithm, Constrained Random Alteration of Network Edges (CRANE), that samples weighted networks with fixed node strengths while retaining the underlying gene regulatory structure. Fixing the node strengths preserves the module resolution while creating more realistic variance in edge weights and reducing bias from promiscuous hub TFs and genes that seed modules associated with disease-independent housekeeping processes (see section “Materials and Methods” for details). We found that CRANE is better able to mimic the “true” null distribution of differential modularity scores arising from subsampled PANDA networks than the configuration model and SBM (Figures 3A,B). Similarly, CRANE better estimates the edge weight variance in WGCNA networks than the configuration model (Supplementary Figure 1). In particular, the mean of the CRANE-generated distribution remains in close proximity to the “true” subsampled null distribution, while other generative models have large deviations across multiple moments of the distribution.

The magnitude of the perturbations in the network created by CRANE is governed by a user-defined parameter α. To choose this parameter appropriately, we compared the properties of networks generated with different α values with the subsampled networks from the previous section. We focused on the distribution of differential modularity scores, the variance in edge weights, and the similarity in community structure (as measured by the normalized mutual information, or NMI) between the original network and the randomized networks. As expected, increasing the parameter α leads to decreasing NMI score (or similarity) (Figure 3C) and increasing edge weight variance (Figure 3D), but there is no single value of α that exactly mimics the subsampled networks (Figure 3B). We decided to use a range of values (α from 0.1 to 0.4) that provide a reasonably good fit to the breast cancer patient data and test the robustness and sensitivity to the exact value below. However, other values of α may be more appropriate in other contexts, depending on the uncertainty in gene expression data and expression correlations.

### Using CRANE to Identify Simulated Disease Modules

We tested whether CRANE could find artificially created disease modules in settings resembling real weighted biological networks. To simulate the effect of measurement noise, we created two independent sets of randomly subsampled (*n* = 50) gene expression data from the same baseline condition, estrogen receptor negative (ER−) breast cancer (BC), and used them to infer two gene regulatory networks, BCN1 and BCN2. Keeping BCN1 as the baseline network, an artificial disease module was created in BCN2 by increasing the edge weights between randomly selected subsets of transcription factors and genes, ranging from 3 to 20 TFs in size, and five times as many genes. We then applied a large panel of methods – namely, ALPACA, consensus clustering, random edge weight permutation, SBM, CRANE, and subsampling – to find differential modules and rank the nodes according to either their differential modularity score (DMS), or by the *p*-value representing how much their scores deviate from the generated null distribution. A Wilcoxon rank-sum test was used to evaluate how highly each method ranked the genes in the true disease module. In order to include the configuration model in this panel, we also performed a second test after applying an exponential transformation on the network edge weights, since the configuration model requires positive edge weights (Gabrielli et al., 2019).

**FIGURE 4:**
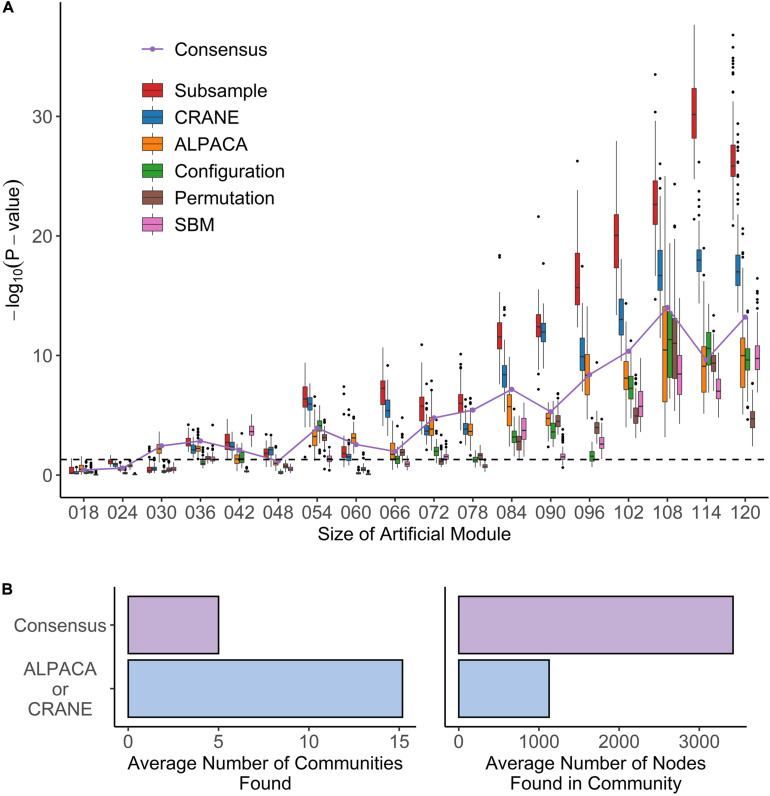
Performance of seven methods on identifying artificial modules in simulated disease networks. **(A)** Box plot shows performance of each method – subsample, CRANE, ALPACA, configuration model, permutation, or SBM – on network simulations with exponentially transformed edge weights. *P*-values (*y*-axis) for various sizes of artificial modules (*x*-axis) are computed using Wilcoxon rank-sum test. **(B)** The boxplots show the total number of modules and module size across all simulation trials for ALPACA versus consensus clustering.

We found that, although ALPACA by itself can successfully recover artificial modules of size greater than 48 nodes (Figure 4A), CRANE was able to dramatically improve performance, as indicated by more significant Wilcoxon *p*-values, showing that the simulated “disease” genes were ranked higher by CRANE than by ALPACA; CRANE also increased F-scores computed using the top 1% ranked nodes in each method (Supplementary Figure 2). Consensus clustering improved performance in recovering a single added module but embeds the artificial module within a much larger community, reducing the resolution (Figure 4B), whereas CRANE maintained high resolution. By the same metrics, CRANE was more successful than random edge weight permutation, the configuration model, and the SBM. This performance gain in CRANE was preserved across α-parameter values ranging from 0.1 through 0.4, suggesting that, within this range, the exact value of α is not critical (Supplementary Figure 3). As expected, the “true” subsampled distribution performed best out of all the methods. We observed a similar trend in performance whether or not the exponential transformation was applied to the network edge weights (Supplementary Figure 4).

### Applying CRANE to Cancer Data

To determine if CRANE can be used to increase the detection of network alterations in complex diseases, we applied CRANE to real biological data. Since there is no “ground truth” dataset for disease modules in transcriptional networks, there is no straightforward way to count false positives and false negatives and compute the precision and accuracy of our results. Instead, we quantified the extent to which highly ranked genes from CRANE are statistically enriched in biological functions driving two well-understood disease processes – angiogenesis in ovarian cancer and estrogen response in breast cancer – using Fisher’s exact test *p*-values. Using our simulation study as a guide (Supplementary Figure 3), we chose α = 0.1 as a value that would provide the biggest performance increase with the least amount of computational cost (the run time of CRANE increases with α due to the deviation correction step).

#### Ovarian Cancer

Ovarian cancer is one of the leading causes of death among women in the developed world (Arend et al., 2013; Bowtell et al., 2015; Reid et al., 2017). Ovarian cancer is divided into many histologic subtypes based on cellular origin, pathogenesis, molecular alterations, and gene expression (Reid et al., 2017). In particular, an angiogenesis gene signature can categorize ovarian cancer patients into a poor-prognosis subtype (Bentink et al., 2012). To test CRANE on angiogenic ovarian cancer, we first applied PANDA to infer ovarian cancer gene regulatory networks from Pan-Cancer TCGA RNA-seq data (Cancer Genome Atlas Research Network, 2011). Normalized RNA-seq profiles were classified into 124 angiogenic and 166 non-angiogenic ovarian cancer tumors as described in Glass et al. (2015). We then applied the same panel of methods as above, ranked the top-scoring genes, and evaluated their functional enrichment for biological processes.

**FIGURE 5:**
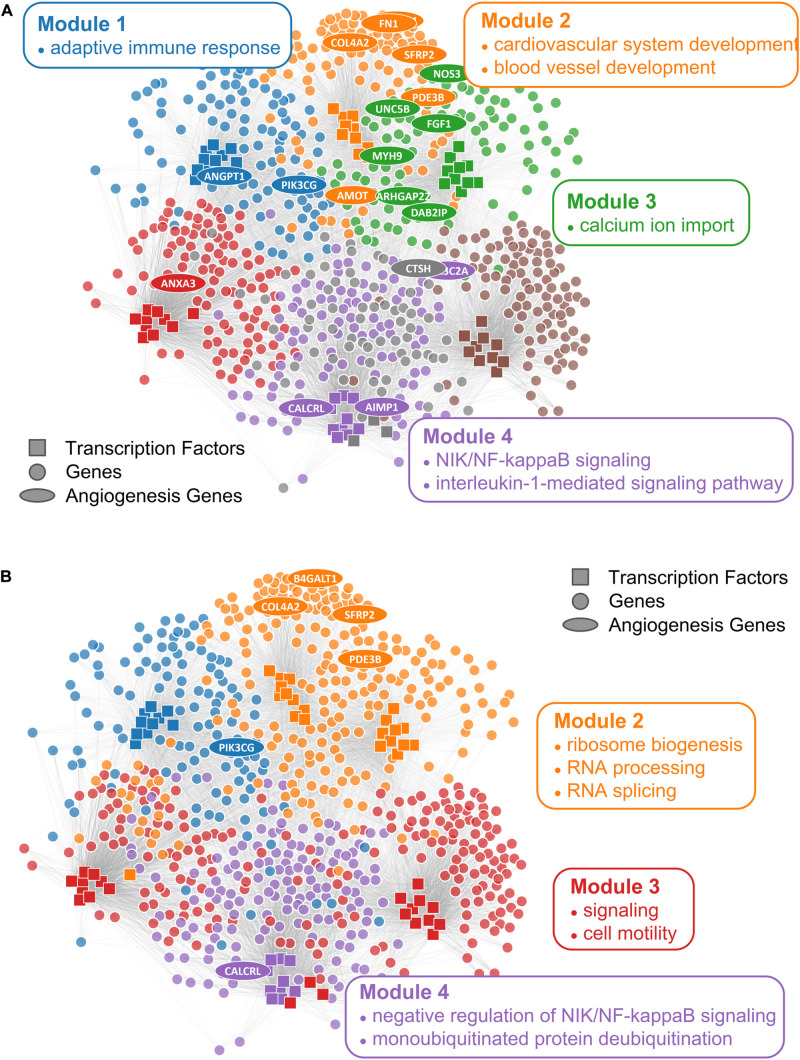
Comparison of ALPACA modules and consensus clustering in angiogenic ovarian tumors. Network with **(A)** ALPACA solution has seven modules while **(B)** the consensus clustering results in four modules. Top 10 core TFs and 100 core genes were extracted from each module based on DMS from ALPACA. The consensus community or ALPACA membership was then overlaid on top by coloring the nodes. The angiogenesis genes (ellipse) were labeled based on whether they were ranked within the top 100 genes in the respective methods. Network is annotated with representative enriched GO terms in each module with *P*_*adj*_ < 0.05.

We first checked the performance of consensus clustering compared to ALPACA to see how the reduction in community resolution would impact the biological interpretation. Consistent with our previous work, ALPACA discovers finer community structure enriched for GO terms that are specific to the angiogenic ovarian cancer phenotype such as “blood vessel development” and “cardiovascular system development” (Figure 5A) (Padi and Quackenbush, 2018). In comparison, consensus clustering results in loss of community resolution (Figure 5B), which in turn leads to the lack of enrichment in more specific GO terms. Instead, communities are enriched for general processes such as “RNA splicing,” “monoubiquitinated protein deubiquitination,” “translation initiation,” and “ribosome biogenesis” (Supplementary Table 1).

**FIGURE 6:**
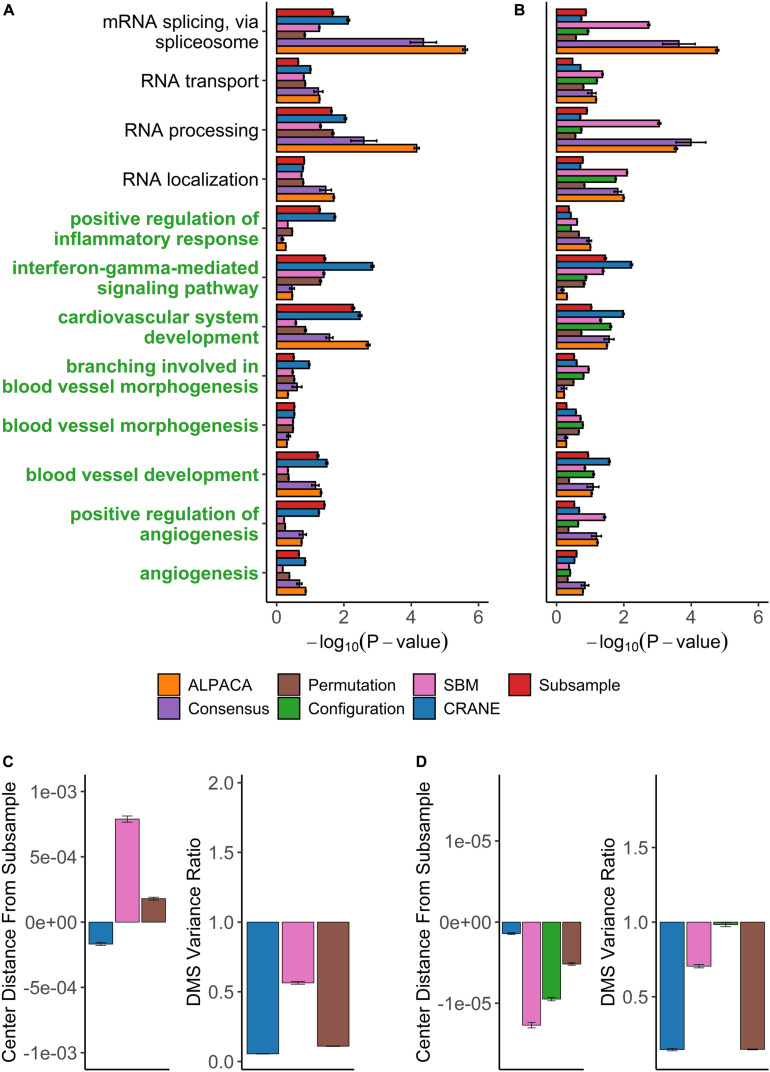
Performance of seven methods on discovering disease-relevant modules associated with angiogenic ovarian tumors. The top five-hundred genes in each module discovered by each method were extracted and subjected to GO term enrichment analysis. **(A,B)** Horizontal bar plots show a curated set of GO terms and their average –log_10_*P*-values over 100 different ALPACA runs. The GO terms (*y*-axis) colored in green are disease-relevant terms while black terms represent general biological processes. **(C,D)** The left vertical bar plot shows the average center distance and the right bar plot shows the average ratio between the mean of the null distribution created from subsampled networks and the mean of the null distribution generated from the indicated methods. Negative distance indicates that the specific method underestimates the center of the “true” subsample distribution. For the variance ratio, values less than 1 represent greater variance in the subsample distribution compared to the indicated methods. The GO term enrichment analysis and the distribution analysis were performed on networks with either **(A,C)** PANDA edge weights or **(B,D)** exponentially transformed edge weights. The error bars represent mean ± S.E.M.

We then applied CRANE (α = 0.1) and found that it showed good performance and resolution in recovering disease-specific processes (Figure 6A). CRANE-ranked genes were statistically enriched for expected GO terms such as “angiogenesis” and “positive regulation of angiogenesis,” with *p*-values similar to the “true” subsampled distribution, and exhibited mild improvement (i.e., more significant Fisher’s exact test *p*-values) over ALPACA (Figure 6A). Although the improvement in GO term detection in the individual modules was modest, we noticed that both CRANE and subsampling increased the ranking of “blood vessel development” related genes, when genes were ranked across the whole network instead of in a module-specific manner (Supplementary Figure 5). This is because the blood vessel development genes are split across two modules which allows them to be masked by other enriched processes present in the same modules, such as inflammation pathways (Supplementary Figure 6). Interestingly, compared to ALPACA and consensus clustering, CRANE reduces signals from non-specific housekeeping processes, like “RNA transport” and “RNA processing.” The permutation and SBM methods performed poorly in uncovering the disease-specific GO terms, as these methods had a tendency to overestimate the DMS distribution while underestimating the variance (Figure 6C).

CRANE and subsampling also consistently identified communities that represent inflammation and immune response. Genes in Module 1 deemed most significant by CRANE were enriched for interferon response, interleukins, cytokine signaling, and inflammation, consistent with the theory that chronic inflammation is associated with risk of cancer (Hanahan and Weinberg, 2011) (Supplementary Table 2). Specifically, immunomodulators and interferon gamma have been proposed as a therapeutic target in ovarian cancer (Wall et al., 2003; Cohen et al., 2016). The enrichment in inflammation and immune response was not readily detectable using ALPACA, permutation, and SBM (Figure 6A and Supplementary Tables 4–6). CRANE is therefore able to uncover additional communities enriched with processes relevant to the disease phenotype.

We also tested our methods after exponentially transforming the edge weights and found that neither CRANE nor the “gold standard” subsampling method improve the recovery of angiogenesis related processes compared to ALPACA (Figure 6B). The exponentiation process leads to a change in community structure in the PANDA networks (NMI = 0.69) that results in most of the blood vessel development genes being concentrated in a single giant differential module (Supplementary Figure 7). The embedment of the angiogenesis genes in a large module along with overall increase in edge weight variance leads to reduction in CRANE performance, whereas other methods have inflated node *p*-values due to a tendency to underestimate the null distribution (Figure 6D).

#### Breast Cancer

Breast cancer is the second most common cancer and a leading cause of death for women worldwide (Bray et al., 2018). Although breast cancer is highly heterogeneous, one of its most important risk factors is overexpression of the estrogen receptor (ER+) leading to increased cell growth (Garcia-Closas et al., 2008; Ahmed et al., 2009; Dunning et al., 2009). Cellular networks in ER+ breast tumors should therefore exhibit increased estrogen signaling.

**FIGURE 7:**
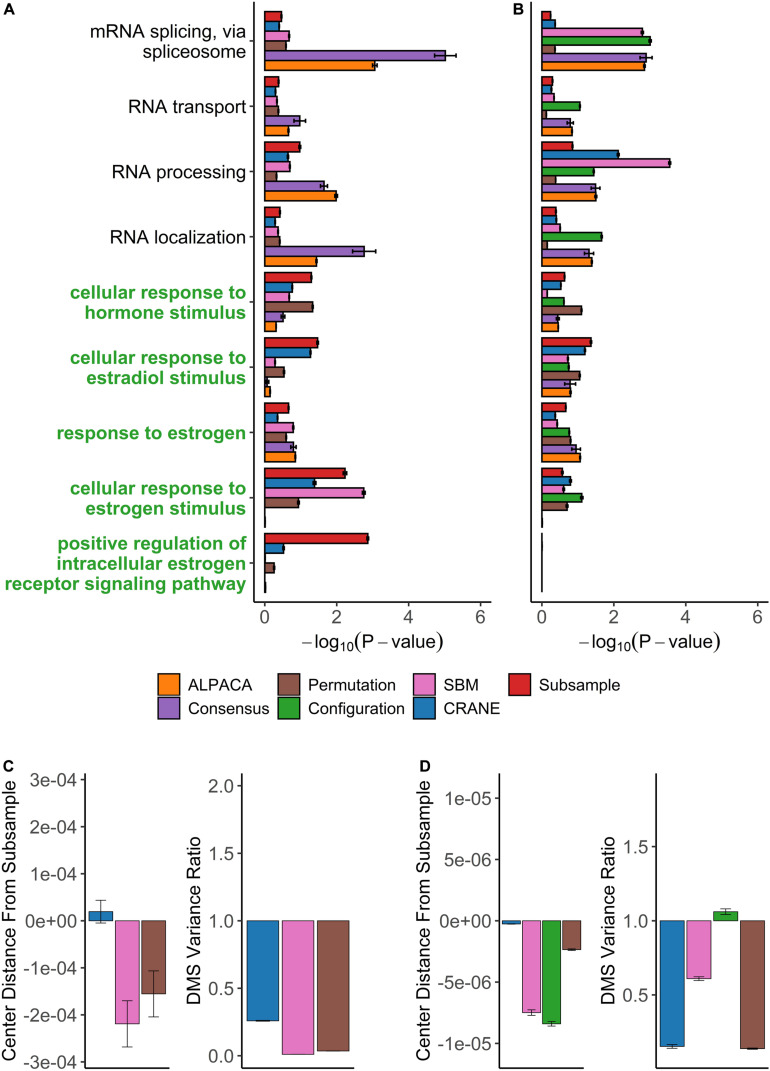
Performance of seven methods on discovering disease-relevant modules associated with ER-positive breast tumors. The top five-hundred genes in each module discovered by each method were extracted and subjected to GO term enrichment analysis. **(A,B)** Horizontal bar plots show a curated set of GO terms and their average –log_10_*P*-values over 100 different ALPACA runs. The GO terms (*y*-axis) colored in green are disease-relevant terms while black terms represent general biological processes. **(C,D)** The left vertical bar plot shows the average center distance and the right bar plot shows the average ratio between the mean of the null distribution created from subsampled networks and the mean of the null distribution generated from the indicated methods. Negative distance indicates that the specific method underestimates the center of the “true” subsample distribution. For the variance ratio, values less than 1 represent greater variance in the subsample distribution compared to the indicated methods. The GO term enrichment analysis and the distribution analysis were performed on networks with either **(A,C)** PANDA edge weights or **(B,D)** exponentially transformed edge weights. The error bars represent mean ± S.E.M.

We used PANDA to infer ER+ (1449 subjects) and ER− (445 subjects) gene regulatory networks from microarray data collected by the METABRIC consortium (Curtis et al., 2012; Pereira et al., 2016). We compared the ER+ network to the ER− network using the same panel of methods as before, and we analyzed the top-ranked genes from each method for enrichment in GO terms. Consensus clustering and ALPACA both failed to detect estrogen-specific pathways (Figure 7A and Supplementary Tables 6, 7). Similar to the results from ovarian cancer, general biological processes such as RNA localization, mRNA splicing, protein catabolic process, and chromosome organization were highly enriched after consensus clustering (Supplementary Table 6).

On the contrary, reranking the nodes using CRANE (α = 0.1) effectively uncovered estrogen specific GO terms such as “cellular response to estrogen” and “positive regulation of intracellular estrogen receptor signaling pathway” with more significant *p*-values than ALPACA, consensus, and the permutation method (Figure 7A and Supplementary Table 8). Similar to the ovarian cancer analysis, CRANE decreased the significance of non-specific housekeeping processes. The “true” subsampled distribution performed even better than CRANE, reinforcing our hypothesis that real disease pathways are robust relative to the underlying noise in regulatory networks.

We also applied the full panel of methods on exponentially transformed breast cancer PANDA networks. The exponential transformation decreased the discovery of estrogen related processes compared to the non-exponentiated network (Figures 7A,B). Nevertheless, all methods showed improvements in the significance level of “cellular response to estrogen stimulus” compared to ALPACA and consensus clustering. The configuration model again had a tendency to underestimate the null distribution of differential modularity scores (DMS), leading to a general inflation of GO term significance (Figure 7D). The permutation method performed well in discovering estrogen-related GO terms. This is likely because for this specific dataset, edge permutation produces a DMS null distribution close to the subsampled distribution. However, over all the analyses we performed, the configuration model, SBM, and permutation methods generally exhibited larger deviations from the subsampled distribution than CRANE, leading to unreliability in their performance (Figures 6C,D, 7C,D). In summary, we found that different generative models may be useful in specific networks, contexts, and conditions, but only CRANE provides reliable and consistent performance across multiple settings in identifying genes statistically enriched for disease-related processes rather than housekeeping functions.

## Discussion

Phenotypic transitions like disease are often driven by the appearance of new groups of genes, or communities, that carry out relevant cellular processes. However, most methods for detecting these new communities rely on maximizing a modularity-based score, and there is no easy way of determining whether the solutions represent true disease modules or whether they could have appeared in healthy tissue due to measurement noise. Consensus clustering offers an effective way of finding stable communities; however, the loss of community resolution leads to a reduction in interpretability. Comparing disease modules with randomized versions of a matched control network could help identify genes that are significantly associated with disease. However, available network generative models are unable to randomize gene regulatory networks while properly controlling the sparsity and edge weight variance. Additionally, biological experiments are resource-limited and do not typically generate enough data to empirically estimate network variance for statistical testing.

We therefore devised CRANE, an algorithm for generating more realistic null distributions of gene regulatory networks by maintaining node strengths and the underlying “hard-wired” structure. We compared CRANE against a “true” null distribution created by down-sampling a large breast cancer dataset to make multiple independent replicate networks. The strength parameter α in CRANE can be used to alter the variance in the edge weights, community structure, and modularity score of the randomized networks. However, our analysis showed that there is no single value of α that fully recapitulates the “true” null distribution. This may be because CRANE independently perturbs all edges while subsampled networks retain correlations between network edges. When applied to cancer networks, CRANE was more accurate at reproducing the center of the subsampled distribution but less accurate at reproducing the variance (Figures 6C,D, 7C,D). We hypothesize that better modeling of the variance of the null distribution would further improve the performance of CRANE.

We used simulated networks with artificial disease modules to evaluate the accuracy and statistical significance of the ranking of disease genes by CRANE. CRANE was consistently more successful in identifying the real disease genes than network generative models and edge weight permutation (Figure 4A). This is likely due to the stricter constraints in CRANE that ensure the randomized networks mimic the original network structure, while other methods deviate due to their looser constraints (Figures 3A,B). We note that the “true” subsampled distribution performed best out of all the methods, suggesting that there is room to further improve CRANE’s ability to capture all the properties of gene regulatory networks.

CRANE also achieved more robust discovery of disease specific processes in cancer regulatory networks. Comparing angiogenic to non-angiogenic ovarian tumors, we found that CRANE leads to a mild improvement in detecting differences in expected pathways like blood vessel development and inflammatory processes. Additionally, CRANE was able to minimize noise from housekeeping processes that are present in all living cells. Comparing ER+ to ER− subtypes of breast cancer, we found that running a modularity maximization method like ALPACA or consensus clustering failed to identify expected changes in estrogen signaling. In contrast, ranking genes by their significance using CRANE revealed that estrogen-related modules were robustly activated in ER+ breast cancer.

The superior performance of CRANE in breast cancer relative to ovarian cancer is likely rooted in differences in the performance of ALPACA in the two datasets. In ovarian cancer, the angiogenesis genes had high ALPACA scores and re-ranking them by significance did not make a big difference (Supplementary Figures 6, 8); in breast cancer, the estrogen genes had lower ALPACA scores to begin with, providing CRANE with more room for improvement. We also found that CRANE performs poorly after exponentiating edge weights, because the exponential transformation leads to a reduction in ALPACA resolution (Supplementary Figures 7, 9). Therefore, exploring other edge weight transformation methods that retain finer community structure while controlling the influence of negative (low-confidence) PANDA edge weights may improve the performance of ALPACA and CRANE.

CRANE assisted differential network analysis minimally requires the user to provide (i) a pair of disease and control networks, and (ii) a list of nodes that defines a candidate module. Although we have applied it in conjunction with PANDA and ALPACA, other network inference and module identification algorithms could also be used in principle. CRANE is designed for weighted networks, with approximately normally distributed edge weights, that incorporate sparsity; in general, network inference methods that use a combination of data-driven correlations and prior information, partial thresholding, or other constraints could be compatible with CRANE. Binary networks with edges either present or absent – e.g., protein–protein interactions measured by IP-MS or Y2H – may require a different statistical treatment. The user-defined candidate module should be more strongly interconnected (higher total edge weight) in the disease network than in the control network, but otherwise could be identified using any method.

In summary, CRANE is a flexible algorithm that can be applied to both weighted unipartite (e.g., WGCNA) and bipartite (e.g., PANDA) gene regulatory networks to generate biologically realistic null distributions. We have demonstrated that this null distribution can be used to better rank the genes that significantly drive disease pathways. In the future, we anticipate that CRANE could be used to evaluate the significance of other features (e.g., information flow or betweenness centrality) of disease networks that are built around a “skeleton” of prior information, like TF binding sites or interaction databases. As gene regulatory networks become an increasingly common framing device for multi-omics data, CRANE provides a robust approach to identify what aspects of these networks are truly altered in disease.

## Data Availability Statement

Publicly available datasets were analyzed in this study. This data can be found here: https://www.cbioportal.org.

## Author Contributions

MP and JL conceived of the project. JL developed the algorithm, performed analyses, and wrote the manuscript. CC performed comparison with network generative models. AG and MP helped to refined the analyses. All authors helped to writing the manuscript.

## Conflict of Interest

The authors declare that the research was conducted in the absence of any commercial or financial relationships that could be construed as a potential conflict of interest.

## Footnotes

1 github.com/PadiLab/ALPACA

2 https://github.com/PadiLab/CRANE

3 https://github.com/PadiLab/ALPACA

## Glossary of Stand-Alone Methods

*PANDA (network inference algorithm)*: infers a weighted bipartite gene regulatory network by iteratively integrating target gene co-expression with transcription factor binding site occurrence (Glass et al., 2013).

*WGCNA (network inference algorithm)*: constructs weighted networks that obey the scale-free topology criterion using soft thresholding of gene correlations (Zhang and Horvath, 2005).

*ALPACA (network analysis method)*: finds candidate disease modules by optimizing a differential modularity score defined as the difference in edge density between disease and matched control networks (Padi and Quackenbush, 2018).

*CONDOR (network analysis method)*: algorithm for detecting community structure in weighted bipartite networks (Platig et al., 2016).

*CRANE (network randomization algorithm)*: perturbs network edges while fixing node strengths and maintaining realistic features of gene regulatory networks.

*Configuration model (network randomization algorithm)*: an exponential random graph model for generating networks from a given node degree or strength sequence (Gabrielli et al., 2019).

*SBM (network randomization algorithm)*: a generative model that defines a probability distribution between node pairs by assuming pre-existing densely connected communities or “blocks” (Aicher et al., 2015).
