# Supplementary figures and images for: Generating Ensembles of Gene Regulatory Networks to Assess Robustness of Disease Modules
Source: Front Genet. 2021 Jan 14;11:603264. doi: 10.3389/fgene.2020.603264 (PMC7841433; doi:10.3389/fgene.2020.603264)

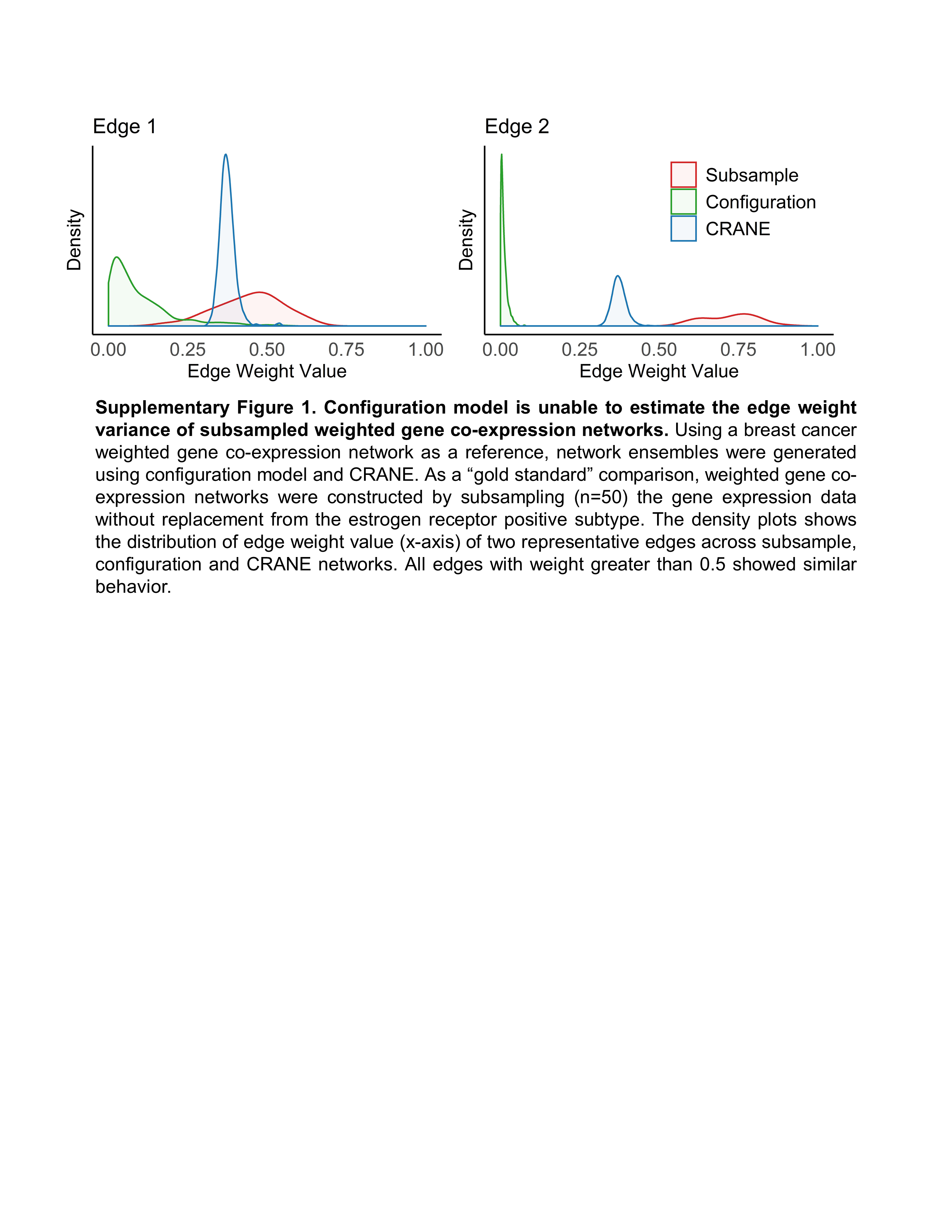

Supplement: Supplementary file 2 [file Image_1.TIF]

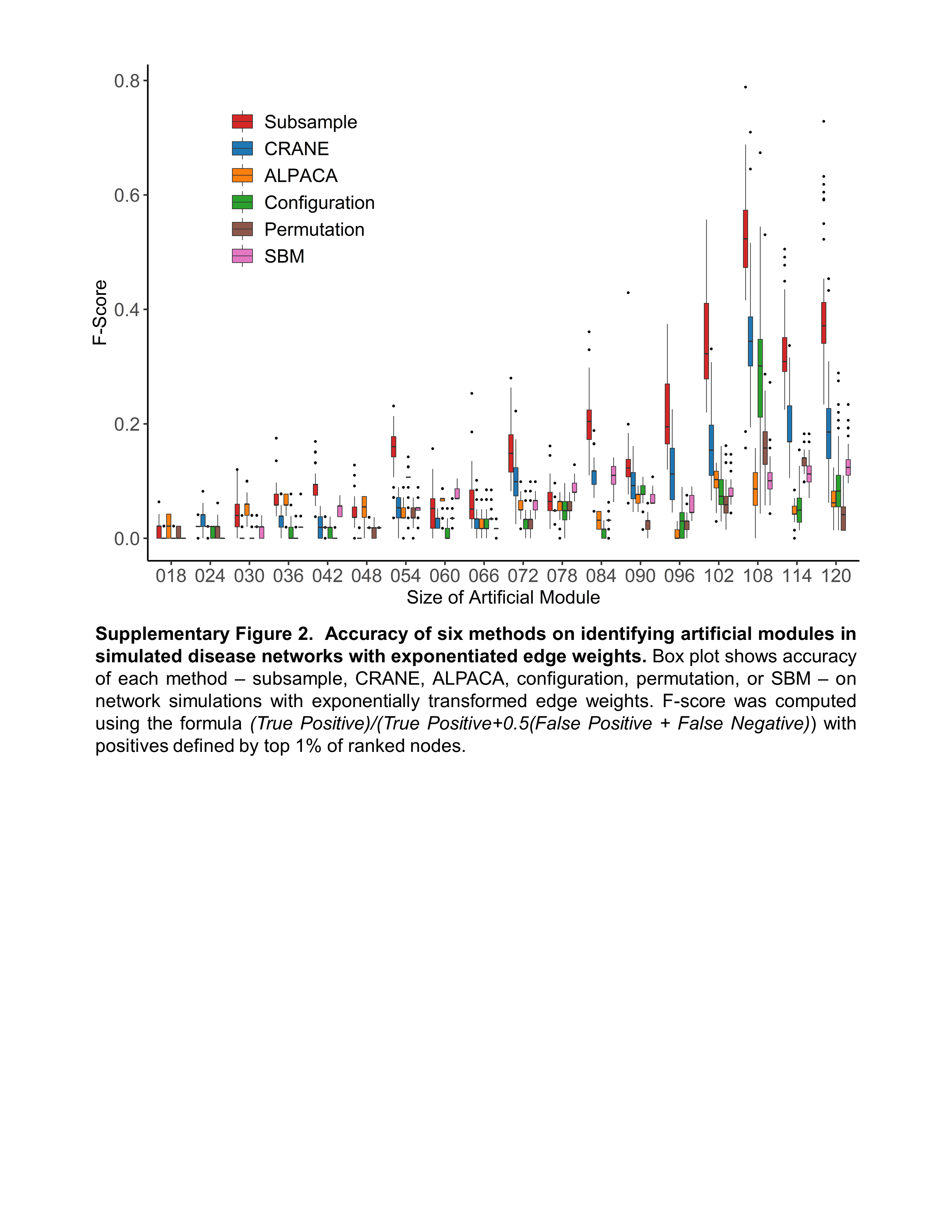

Supplement: Supplementary file 3 [file Image_2.TIF]

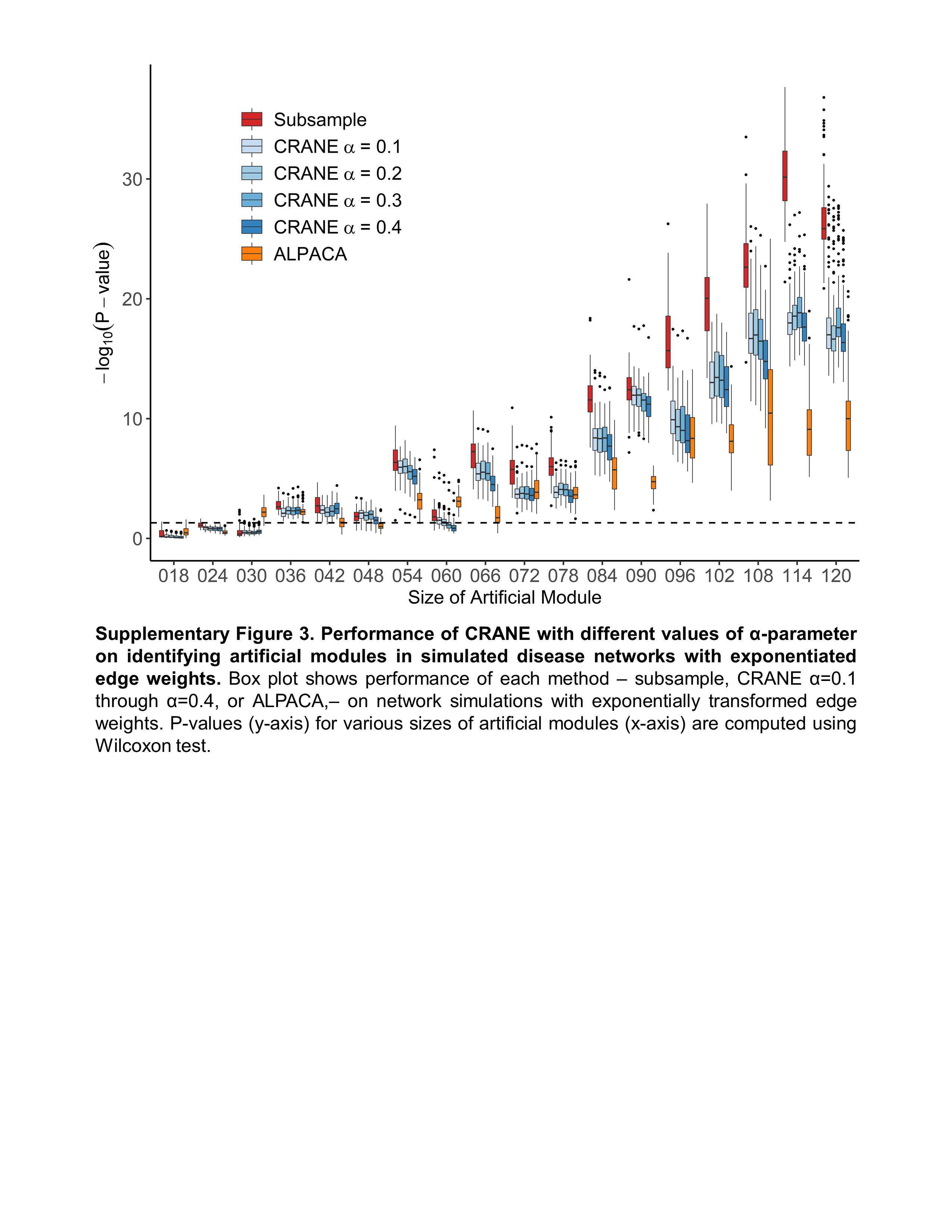

Supplement: Supplementary file 4 [file Image_3.TIF]

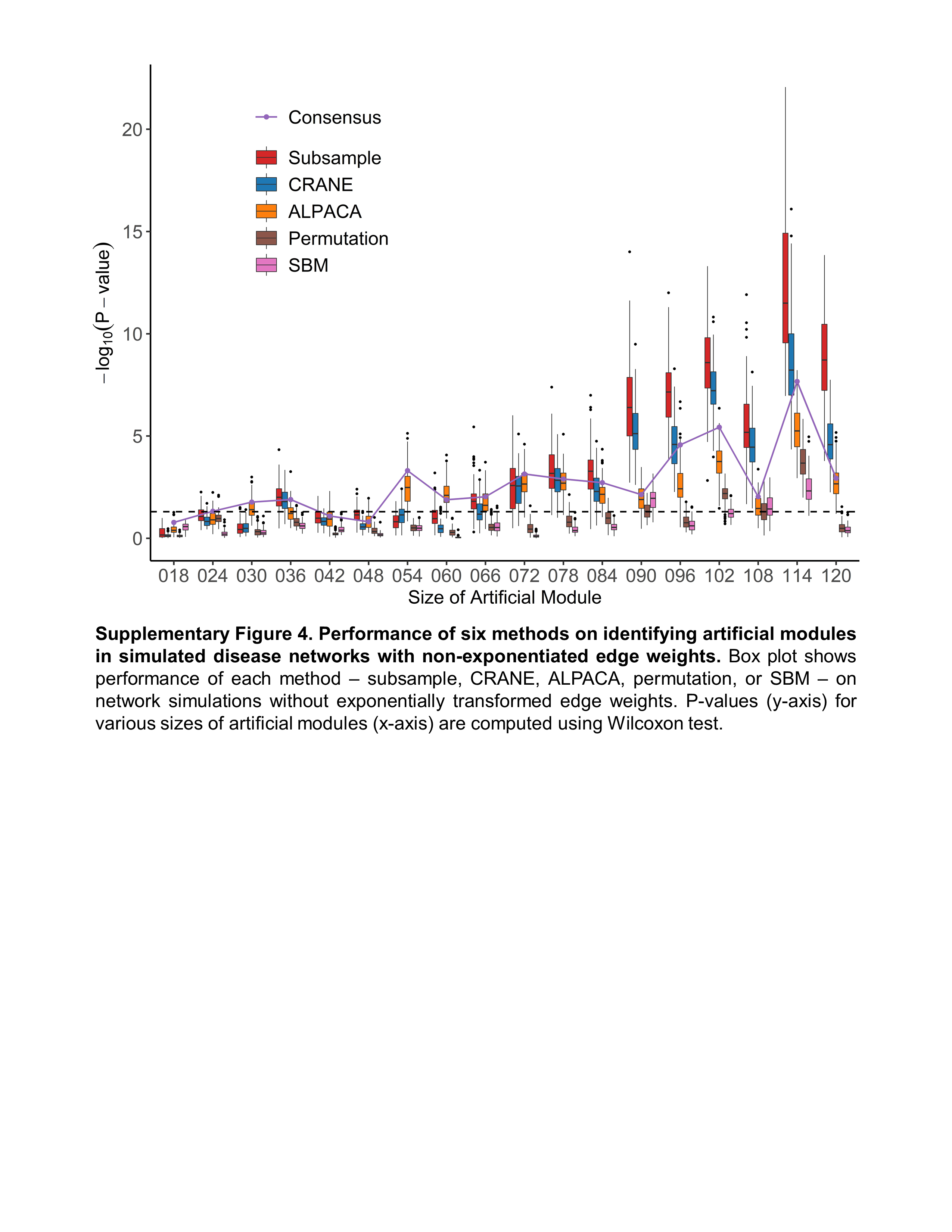

Supplement: Supplementary file 5 [file Image_4.TIF]

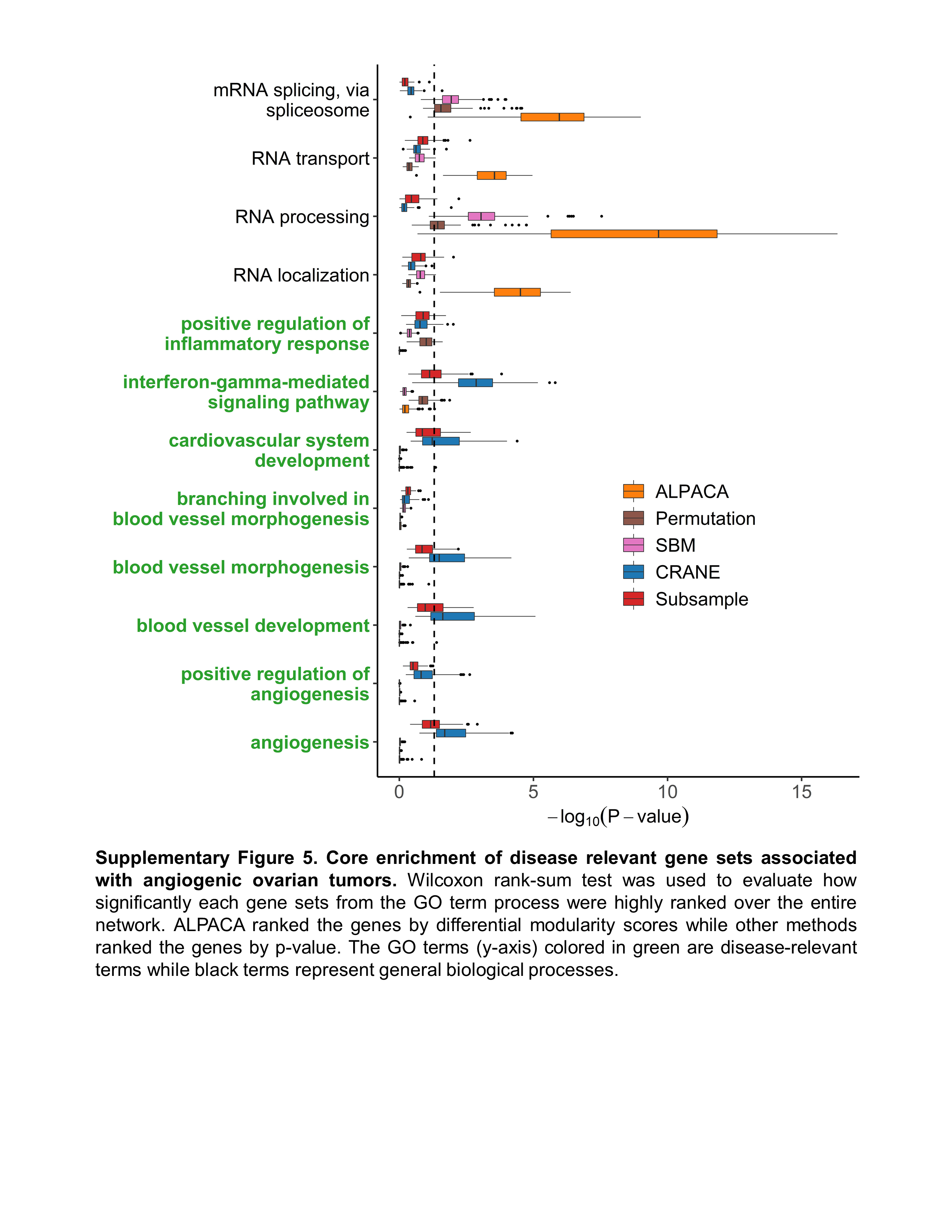

Supplement: Supplementary file 6 [file Image_5.TIF]

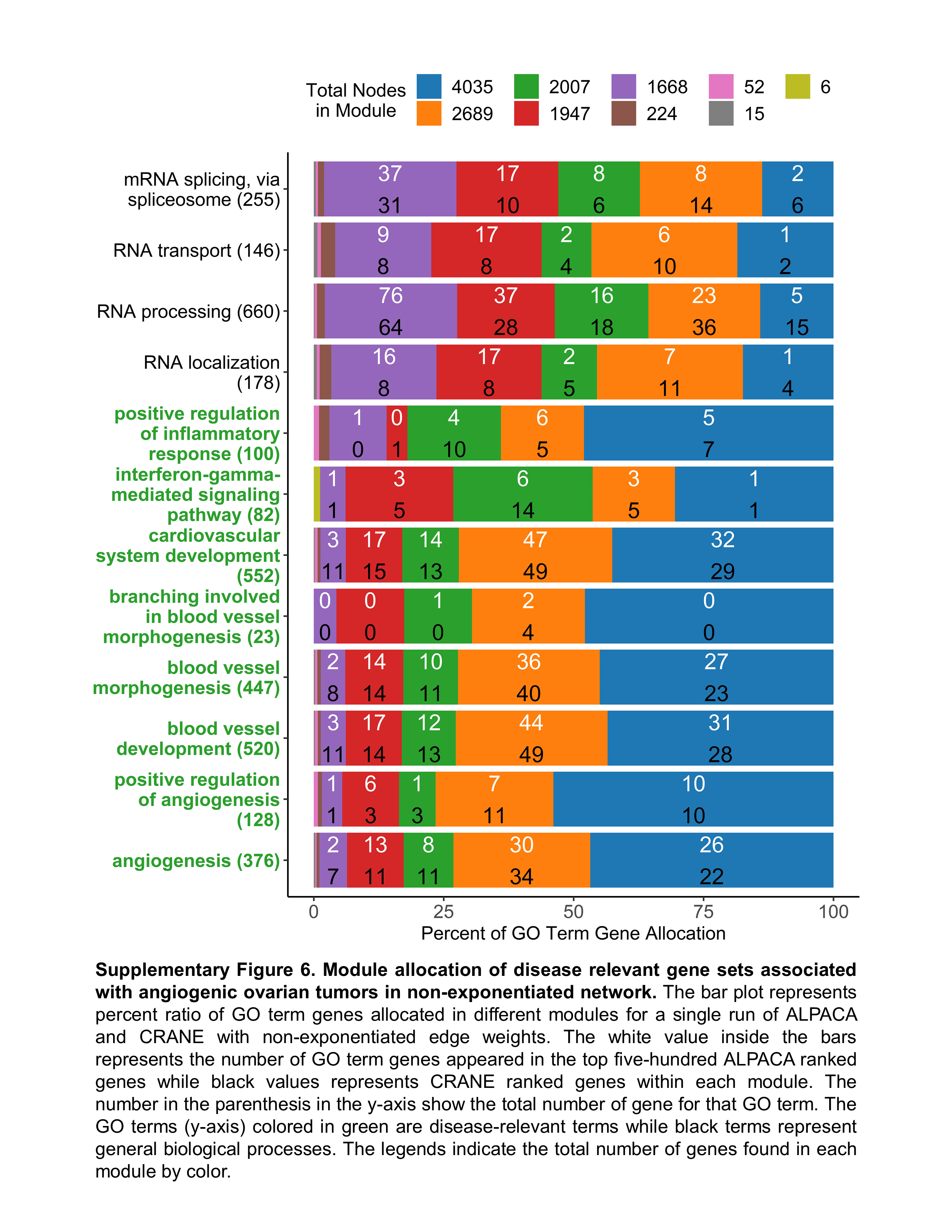

Supplement: Supplementary file 7 [file Image_6.TIF]

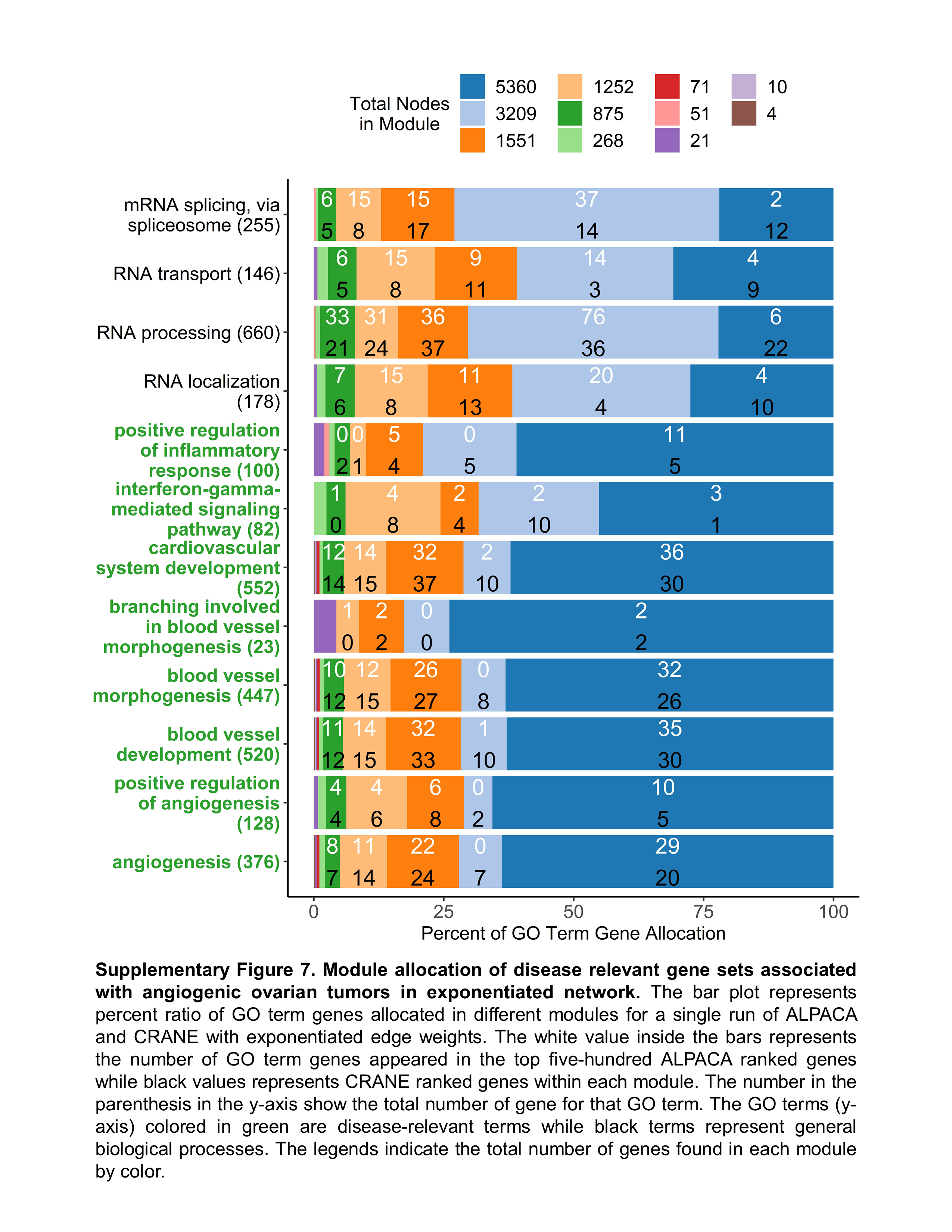

Supplement: Supplementary file 8 [file Image_7.TIF]

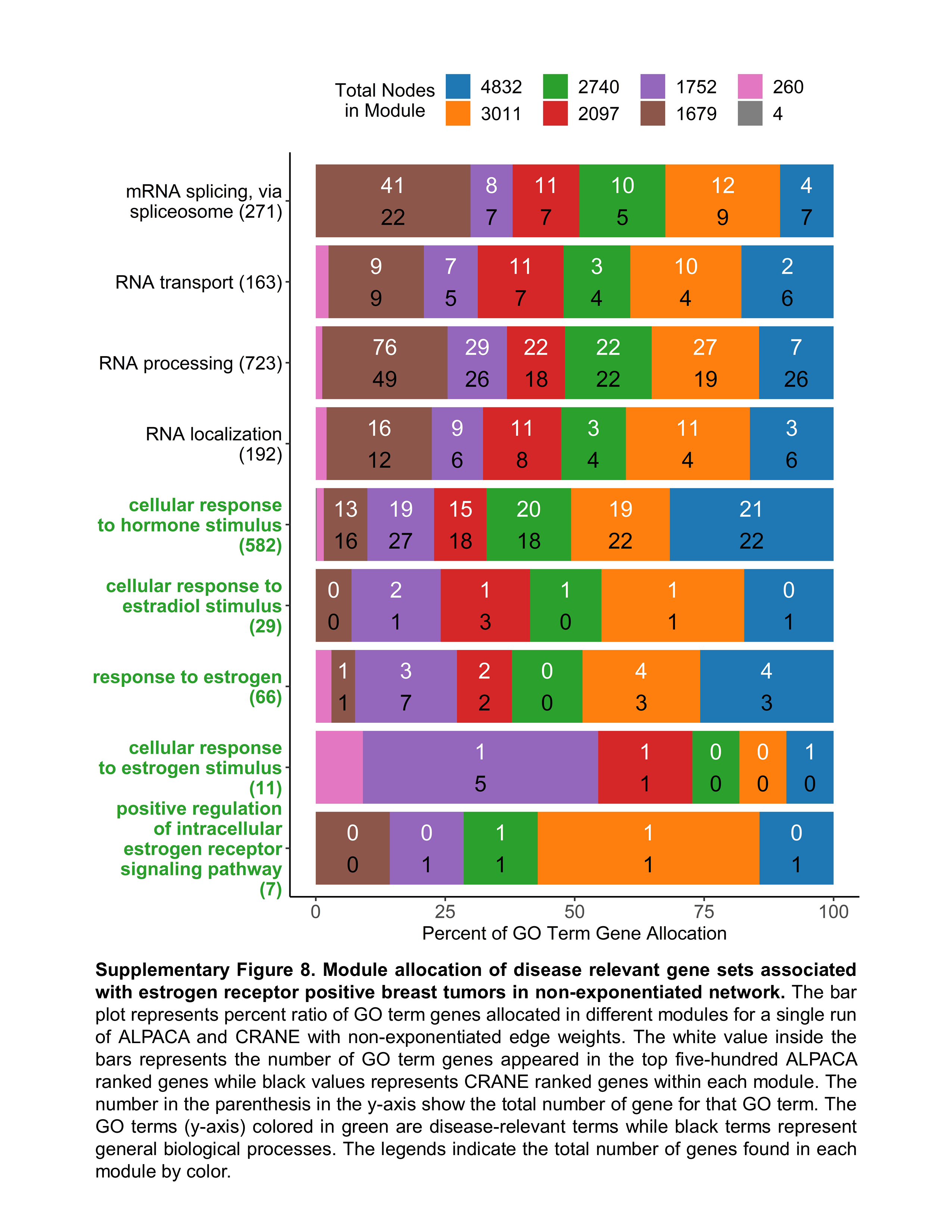

Supplement: Supplementary file 9 [file Image_8.TIF]

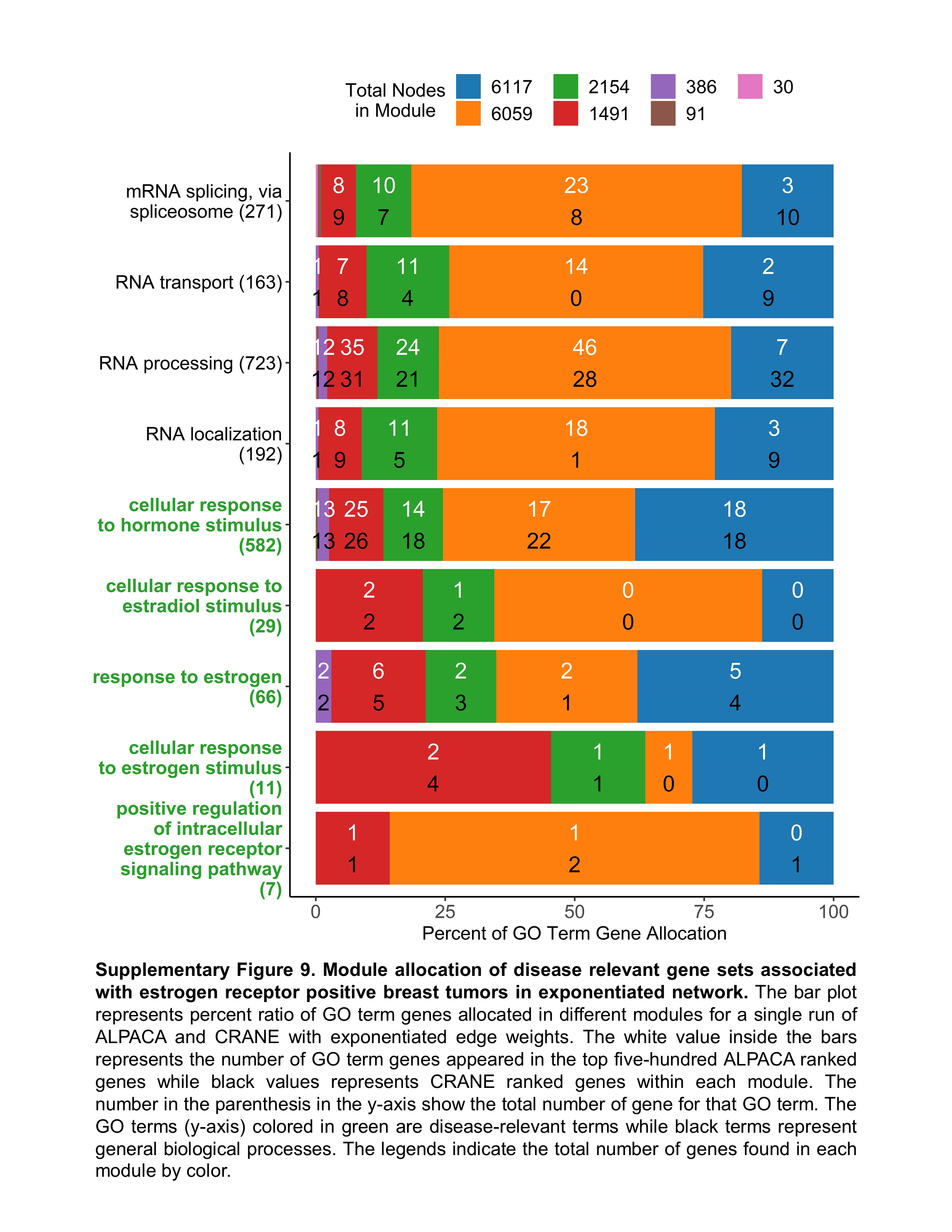

Supplement: Supplementary file 10 [file Image_9.TIF]
